# Vitamin K and women's health: a review

**DOI:** 10.3389/fgwh.2025.1590414

**Published:** 2025-07-11

**Authors:** Sharifa AlBlooshi

**Affiliations:** Department of Health Sciences, College of Natural and Health Sciences, Zayed University, Dubai, United Arab Emirates

**Keywords:** vitamin K, women's health, bone health, cardiovascular disease, diabetes, menopause, immune system, cancer

## Abstract

Vitamin K is a fat-soluble vitamin essential in various physiological functions such as blood coagulation, bone metabolism, cardiovascular health, glucose regulation, immune function, neuroprotection, and vascular health. In this narrative review, studies from databases including PubMed, Google Scholar, Scopus, and the institutional database of Zayed University were selected. The role of vitamin K in women's health, with a focus on osteoporosis, postmenopausal health, cardiovascular health, diabetes, cancer, kidney health, brain health, vascular health, and pregnancy were explored. The role of vitamin K in activating vitamin K-dependent proteins is essential for many of its functions. Vitamin K-dependent proteins such as osteocalcin and matrix Gla protein are crucial to many physiological processes such as bone mineralization, vascular calcification, and cardiovascular health. It also modulates glucose metabolism by enhancing insulin sensitivity, reducing oxidative stress, and influencing β-cell function. Vitamin K exhibits anti-cancer properties in cancer research, particularly in breast, cervical, and ovarian cancer models. It also plays a role in brain health including cognitive function, neuroinflammation reduction, and neurodegenerative disease prevention. Similarly, in kidney disease, vitamin K has been linked to chronic kidney disease progression and vascular calcification. Vitamin K's role in pregnancy includes its impact on neonatal coagulation and neurodevelopmental outcomes. Despite the promising role of vitamin K, findings are mixed on its role in bone mineral density and fracture prevention, cardiovascular protection, protection against vascular calcification, diabetes prevention, cancer risk, kidney dysfunction, and its role in maternal and child health. These highlight the need for further research, particularly longitudinal and randomized controlled trials, to determine optimal intake, efficacy of supplementation, and long-term health impacts of vitamin K in women.

## 1 Introduction

Vitamin K is a fat-soluble vitamin found in two forms: phylloquinone (vitamin K1) and menaquinones (vitamin K2) (1). These compounds share a common structure of 2-methyl-1,4-naphthoquinone core (menadione) (2). Vitamin K1 has a phytol substituted chain, while vitamin K2 contains unsaturated isoprenyl side chains, designated as MK-4 through to MK-13, depending on its length (3). Vitamin K1 is relatively stable to heat but highly sensitive to light and should be stored in dark containers (2).

Vitamin K1 (phylloquinone) is the most widely found dietary Vitamin K, constituting 75%–90% of dietary vitamin K intake but has low bioavailability. It is found in sources such as broccoli, Brussels sprouts, kale, spinach, parsley, mint, and certain vegetable oils (e.g., soybean oil) (2). Menaquinones are limited to certain animal products and fermented foods (1). Certain anaerobic bacteria produce it, the amount varying based on the bacterial species, and can be found in fermented foods such as cheese, sauerkraut, and natto (2). Cheese was found to be the most important source of dietary long-chain MKs (MK-8 and MK-9) (3). Vitamin K3, a synthetic hydrophilic form, is an intermediate in human metabolism (2).

The recommended daily intake (RDI) for vitamin K varies regionally. The National Academy of Medicine recommends 120 µg/day for men and 90 µg/day for women, the World Health Organization (WHO) recommends 65 µg/day for men and 55 µg/day for women, and the European Communities recommend 75 µg/day (4). Vitamin K2 intake often requires supplementation as food sources contribute only a small portion of the total intake (4).

Vitamin K is essential for blood coagulation and acts as an essential coenzyme responsible for modifying glutamic acid residues in certain proteins to γ-carboxyglutamic acid residues (1). There are at least 18 or 19 human physiological proteins and 1 pathological protein, known as Gla proteins, for which vitamin K is needed for γ-glutamyl carboxylation post-translation (2). Vitamin K dependent proteins, such as matrix Gla protein (MGP), osteocalcin, and Gas-6 play roles such as preventing tissue calcification, bone metabolism, and cell cycle regulation (1). In addition to acting as an enzyme cofactor, vitamin K plays roles such as having anti-inflammatory effects and acting as a ligand for steroid and xenobiotic receptors (1). In its reduced form, vitamin K exhibits an antioxidative activity 10–100 times higher than that antioxidants such as alpha-tocopherol and ubiquinone. This helps it protect the cellular membrane, proteins, and DNA from oxidative damage (4).

Measuring Vitamin K levels in serum has been a challenge, thus relying on indirect markers like uncarboxylated osteocalcin (5). Vitamin K deficiency is rare in adults but can occur due to poor dietary intake liver disease, cholestasis, cystic fibrosis, alcoholism, malabsorption, bariatric surgery, or certain drugs like coumarin-based anticoagulants, rifampicin, and antibiotics. Infants are at risk of deficiency due to poor vitamin K stores, low levels in breast milk, and immature gut microbiota (2). The symptoms of vitamin K deficiency include the spontaneous appearance of purple or red spots or bruises on the skin, epistaxis (nosebleed), and bleeding from various sites, such as gastrointestinal, genitourinary, and gingival areas (2). Vitamin K deficiency is associated with increased levels of dephosphorylated-uncarboxylated MGP and is linked to chronic diseases such as cardiovascular and kidney diseases (4).

The position of vitamin K in health and disease has been reported including roles in cardiovascular health, bone development, fractures, diabetes development, metastasis of multiple cancer cell lines, renal artery functions, immunosuppression, and protective effects on neurons (4). The role of vitamin K in women's health needs to be understood due to the unique physiological and hormonal changes women experience across different stages of life from pregnancy and lactation to menopause, which influence bone health, cardiovascular health, immune function, and other health outcomes. This review discusses the role of vitamin K on women's health with a focus on bone health and osteoporosis, cardiovascular health, vascular calcification, kidney diseases, insulin sensitivity, diabetes, cancer, immune system and immunity, brain diseases, and its role during pregnancy and postmenopausal health.

### 1.1 The mechanisms of vitamin K1 and K2

Vitamin K exists primarily in two forms relevant to human physiology: vitamin K1 (phylloquinone) and vitamin K2 (menaquinones). Both serve as essential cofactors for the enzyme γ-glutamyl carboxylase, which catalyzes the post-translational γ-carboxylation of specific glutamate residues in vitamin K dependent proteins. This modification is critical for enabling these proteins to bind calcium ions, a prerequisite for their biological activity (6). Vitamin K1, predominantly derived from plant sources, is absorbed in the small intestine and preferentially transported to the liver, where it facilitates the γ-carboxylation of hepatic clotting factors (II, VII, IX, and X) and regulatory proteins (C and S), thereby playing a central role in the coagulation cascade. Its relatively short half-life limits its distribution primarily to hepatic tissues (4).

Vitamin K2 comprises a group of menaquinones varying in side-chain length, produced by bacterial synthesis and present in fermented foods. Unlike K1, K2 has a longer half-life and is transported by low-density lipoproteins, allowing broader distribution to extrahepatic tissues such as bone, vasculature, and the brain. In these tissues, K2-mediated γ-carboxylation activates proteins such as osteocalcin, which regulates bone mineralization, and matrix Gla protein, a potent inhibitor of vascular calcification. Beyond carboxylation, vitamin K2 also influences cellular functions by modulating gene expression and inflammatory pathways, notably through inhibition of nuclear factor-κB (NF-κB) signaling (7). Thus, while both K1 and K2 share a common enzymatic mechanism centered on γ-carboxylation, their differences in absorption, transport, tissue targeting, and non-carboxylative activities underpin their distinct physiological roles (7).

## 2 Vitamin K, osteoporosis, and bone health

Osteoporosis is a non-communicable bone disease that affects 200 million people worldwide (8). One in three women and one in five men above the age of 50 are affected by this condition globally (9). One of the non-modifiable risk factors for osteoporosis is the female sex and it has more prevalence in post-menopausal women due to decreased estrogen levels that negatively impact bone formation by increasing the sensitivity to bone resorption effect of parathyroid hormone (8).

Vitamin K has been shown to have a pivotal role in bone health. Vitamin K is essential in the carboxylation of Vitamin K dependent proteins (VKDP), particularly osteocalcin (OC) and MGP that regulate extracellular matrix mineralization, osteoblast differentiation, and osteoclastogenesis (121). OC is synthesized by osteoblasts and needs Vitamin K for carboxylation to be transformed into its active form to be able to bind calcium and hydroxyapatite in bone, critical for hydroxyapatite crystal formation (5, 10). MGP, another VKDP, inhibits vascular calcification, indirectly supporting bone strength by regulating calcium distribution (5). In addition, vitamin K influences osteoblast differentiation to osteocytes and limits osteoclastogenesis, thereby promoting bone formation and reducing bone resorption (5, 10, 11).

Findings on the impact of vitamin K intake from food and supplementation have shown inconsistency, some suggesting increased bone mineral density (BMD) and decreased fracture risk, while others reported no significant association. A longitudinal study conducted involving 1,347 community-dwelling older Australian women over 14.5 years reported a dose-dependent positive relationship between vitamin K1 intake and physical function, as well as a lowered risk of long-term injurious falls with higher intake (12). Conversely, the study found no significant association between vitamin K2 intake and muscle function or fall-related hospitalization. This highlights that the two forms of vitamin K might have different roles, with K1 having a beneficial effect in this context. An observational cohort study including 1,089 older adults corroborated these findings, showing that higher plasma phylloquinone (vitamin K1) levels (≥1.0 nM) are associated with better physical performance scores and faster 20-m gait speed at baseline over a 4–5-year follow-up (13). The associations were found to be less consistent longitudinally, implying a sustained vitamin K1 status may be needed to sustain health benefits. The study is limited in that it measured vitamin K status at a single point in time, failing to account for changes over time. Further evidence from a similar cohort study showed the combined effects of adequate vitamin K1 and vitamin D levels on improved physical performance (14).

A randomized controlled trial with 30 participants aged 40–74 years conducted over three 4-week experimental phases found that the consumption of green leafy vegetables rich in vitamin K1 significantly reduced serum levels of total and uncarboxylated osteocalcin, showing potential for bone material property improvement (15). The study's short duration, however, limits conclusions about long-term effects on bone density and fracture prevention. Similarly, a prospective cohort study in 1,417 postmenopausal Japanese women revealed that habitual natto consumption, rich in MK-7, was linked to a 44% reduced risk of osteoporotic fractures compared to low intake. Natto intake was associated with higher BMD at the femoral neck and reduced bone loss during the follow-up period (16).

The relationship between vitamin K supplementation and BMD is less conclusive. A double-blind randomized controlled trial (RCT) involving 105 postmenopausal women with osteoporosis found that there were no significant improvements in BMD at the lumbar spine, femoral neck, or total hip in participants with either vitamin K1 or K2 over 18 months (17). Regardless, vitamin K supplementation had a modest effect on structural benefits such as reductions in endocortical diameter, suggesting potential benefits that may help bone stability. Similarly, Bartstra et al. (18) found that six months of vitamin K2 supplementation (360 µg/day) did not halt the progression of arterial calcification or decline in BMD in patients with type 2 diabetes mellitus (T2DM) and a history of cardiovascular disease (CVD) although it reduced inactive MGP. The study's predominantly male cohort and restriction to patients with T2DM limit its applicability to women. Knapen et al. (19) demonstrated that MK-4 supplementation in postmenopausal women aged 55–75 years with no history of metabolic bone diseases or osteoporosis, significantly increased femoral neck BMC and width improving bone geometry and strength, while it had no significant effects on BMD. On the other hand, a randomized double-blind, placebo-controlled intervention trial in dialysis patients showed that Vitamin K (MK-7) supplementation led to site-specific changes in BMD, preventing lumbar spine BMD decrease while accelerating BMD loss at the 1/3 distal radius (20). These findings indicate that MK-7 supplementation has localized effects and does not support general use for bone preservation in dialysis patients. The study is limited in generalizability due to its small sample size and its restriction to dialysis patients.

The inconsistencies in findings related to the effect of vitamin K supplementation on BMD highlight the need for longer-term studies to better understand the effects of vitamin K supplementation on bone health. The difference in effects between vitamin K1 and K2, and the site-specific benefits necessitate the need for further investigation to understand the distinct mechanisms involved. Additionally, the underrepresentation of women in certain studies limits the generalizability to broader female demographics and underscores the need for studies focusing on the female population given females are at a higher osteoporosis risk.

## 3 Vitamin K and post-meonopausal health

Post-menopause is linked to significant health effects due to hormonal and metabolic changes. Body composition change happens during the menopause transition, with fat mass increasing while lean mass decreases (21). Skeletal muscle loss starts before menopause and accelerates during the transition increasing the risk of sarcopenia which is associated with functional disability, falls, fractures, and mortality (22–24). Menopause is considered a female-specific risk factor for CVD (24). The decline in the hormone estradiol levels leads to abnormal lipid metabolism, resulting in increased low-density lipoprotein cholesterol (LDL-C), triglycerides, and total cholesterol. This increases cardiovascular risks such as arterial plaque formation, atherosclerosis, vascular stiffening, and coronary heart disease (22, 23). The decline in estrogen which normally acts as an antioxidant, leads to higher oxidative stress levels, increasing lipid peroxidation, inflammatory cytokine production, and chronic inflammation (22). Reduced estrogen-mediated vasodilation during and after menopause leads to a significant rise in blood pressure, making hypertension the major risk factor for stroke and heart failure in postmenopausal women (23). Additionally, postmenopausal women experience increased visceral fat accumulation in the abdominal region leading to an increased risk of central obesity, metabolic syndrome, and T2DM (22, 23). The higher fasting glucose levels and insulin resistance observed in post-menopausal women pose increased T2DM risk (22, 23). Beyond metabolic and cardiovascular risks, postmenopausal women experience problems such as hot flashes and night sweats, sleep disturbances, depression and anxiety, cognitive performance decline, and genitourinary issues (24). These health effects reduce the quality of life, underscoring the importance of targeted interventions to manage postmenopausal health risks.

Vitamin K plays important roles in postmenopausal health including cardiovascular health, bone health, and metabolic processes. Despite its importance, postmenopausal women are at higher risk of vitamin K2 deficiency (25). A study conducted in postmenopausal women with osteoporosis found that women with higher serum vitamin K1 levels had a significantly lower risk of fractures**,** and improved hip bone strength, even though it had no significant effect on BMD (26). Evidence on the effects of different forms of vitamin K supplementation in osteoporotic and non-osteoporotic post-menopausal women has been mixed. A one-year clinical trial by Jiang et al. (27) found that vitamin K2 (MK-4) reduced undercarboxylated osteocalcin, improved lumbar spine and trochanter BMD, but did not impact femoral neck BMD, fracture risk or fall incidence in postmenopausal women with osteoporosis. Another clinical trial by Binkley et al. (28) observed a similar finding concerning the reduction in undercarboxylated osteocalcin levels with vitamin K supplementation in healthy menopausal women. They reported that there were no significant changes in BMD unlike the study by Jiang et al. (27) that reported the site-specific impact of vitamin K2 on BMD. They also found vitamin K supplementation did not affect bone turnover markers, bone geometry, or strength. Similar to their finding, a three-year clinical trial in post-menopausal women with osteopenia found that vitamin K2 (MK-7) supplementation significantly improved osteocalcin carboxylation but did not prevent BMD loss, alter bone microarchitecture, or improve bone strength (29). In contrast, a 3-year clinical trial by Knapen et al. (30) found that vitamin K2 (MK-7) supplementation slowed BMD loss at the lumbar spine and femoral neck, enhanced bone strength indices, and reduced vertebral height loss in postmenopausal women. However, they did not observe statistically significant impact on fracture risk.

Beyond bone health, vitamin K2, by activating MGP, inhibits calcium deposition in blood vessels which prevents vascular calcification (31). A study by Braam et al. (32) reported that Vitamin K1 and D supplementation preserved arterial elasticity, improved vascular elasticity, and stabilized pulse pressure in post-menopausal women over three years. However, they found that it did not affect endothelial thickening related to atherosclerosis. The protective effects against vascular calcification may be linked to the activation of MGP. Interventional studies, evaluating the isolated effect of vitamin K on CVD in postmenopausal women is lacking. Anti-cancer effects of vitamin K on ovarian and cervical cancer have been suggested in *in vitro* studies but clinical studies in postmenopausal women are lacking (33). Vitamin K has been reported to offer neuroprotection against aging diseases such as Alzheimer's and Parkinson's through its antioxidant and anti-inflammatory effects. It contributes to liver health by slowing hepatocellular carcinoma (HCC) progression, improves metabolic health by enhancing insulin sensitivity and reducing diabetes risk, and lowers inflammation by reducing oxidative stress and cytokines like IL-6 and TNF-α. Vitamin K2 also promotes dental health by maintaining calcium balance in teeth and strengthening enamel (31).

## 4 Vitamin K and immunity

Emerging research has suggested vitamin K may have a significant influence on immunity and immune health. Vitamin K activates vitamin K-dependent proteins (VKDPs) such as Protein S (PROS1) and Growth Arrest-Specific Gene 6 (GAS6) that regulate immune responses and coagulation through TAM receptors **(**TYRO3, AXL, and MERTK) (34). Protein C and protein S exhibit anti-inflammatory and anticoagulant effects, while Gas6 suppresses TNF-α (35). Additionally, Protein S interacts with the C4b-binding protein (C4BP), which modulates immune responses by interacting with B lymphocytes (36).

In the context of infectious diseases like COVID-19, vitamin K inhibits pro-inflammatory cytokines (IL-6, IL-1β, and TNF-α) and enhances the integrity of alveolar-capillary membranes reducing the risk of acute respiratory distress syndrome (ARDS) (37). Furthermore, vitamin K acts as an antioxidant by interacting with ferroptosis suppressor protein 1 (FSP1) preventing lipid oxidation and ferroptosis (iron-dependent cell death associated with oxidative stress and lipid oxidation) (38). In autoimmune diseases, vitamin K plays a crucial role in reducing inflammation and oxidative stress. It supports gut microbiota balance in inflammatory bowel disease, enhances pancreatic β-cell survival, and improves insulin secretion in T1DM, it promotes myelin generation and protects neurons from oxidative damage in multiple sclerosis, and suppresses synovial inflammation in rheumatoid arthritis potentially delaying its progression (39). In Systemic Lupus Erythematosus (SLE), vitamin K influences disease activity through GAS6 and its receptor-mediated regulation of inflammation (36). Additionally, vitamin K2 showed anticancer properties, particularly in bladder and liver cancer cells, where it triggers mitochondrial-mediated apoptosis, through inhibition of NF-κB and oxidative stress pathways (36).

A three-year supplementation trial with 500 µg/day of vitamin K1, by Shea et al. (40) showed that higher plasma phylloquinone (vitamin K1) concentrations were inversely associated with inflammatory markers like IL-6 and C-reactive protein (CRP), and poor vitamin K status was linked to higher levels of inflammatory cytokines. However, vitamin K supplementation did not significantly reduce cytokine levels. The study involved a generally healthy cohort, which may have limited detectable changes in inflammation. Similarly, a cross-sectional study by Shea et al. (40) found that higher plasma phylloquinone (a form of vitamin K1) and phylloquinone intake were inversely associated with lower levels of pro-inflammatory cytokines such as interleukin-6 (IL-6), tumor necrosis factor receptor-2 (TNF-R2), osteoprotegerin, CD40 ligand, and intercellular adhesion molecule-1. Higher dietary intake of vitamin K was also associated with reduced levels of CRP, fibrinogen, myeloperoxidase, and urinary isoprostanes (a marker of oxidative stress) (41).

Additionally, synthetic forms of vitamin K, vitamin K3 (Menadione), and vitamin K4 (Menadiol), have been shown to selectively inhibit the NLRP3, a key player in the immune response that promotes inflammation by activating IL-1β and IL-18 (42). The study showed that vitamin K3 and K4 reduced inflammation in a mouse model of peritonitis by lowering IL-1β secretion and neutrophil infiltration, showing potential for NLRP3-associated inflammatory diseases, such as T2DM, atherosclerosis, Alzheimer's disease, and gout (42). Checker et al. (43) reported vitamin K3 (Menadione) through the suppression of NF-κB and MAPK pathways, reducing macrophage-mediated inflammation, delaying graft-vs.-host disease (GVHD), and inhibiting CD4+ T-cell homeostatic proliferation modulates immune responses by altering cellular redox status, suppressing lymphocyte proliferation, and inhibiting T-cell activation and cytokine production showing potential as an immunosuppressive agent. However, the findings by both Zheng et al. (42) and Checker et al. (43) are based on mouse models and cell cultures, and their application is limited to human subjects. There are also toxicity concerns with vitamin K3 such as liver damage and hemolytic anemia which must be considered before clinical application.

## 5 Vitamin K and cardiovascular health

Vitamin K has been implied to have a role in cardiovascular health including the regulation of vascular calcification, atherosclerosis, and overall cardiac function. In a cross-sectional health examination study involving 4,092 Danish adults, higher dp-ucMGP levels, an indication of low vitamin K status, were associated with increased cardiovascular risk (44). A prospective study by Bellinge et al. (45), which included 53,372 Danish individuals aged 50–65, found that vitamin K intake was strongly associated with atherosclerotic cardiovascular disease (ASCVD) risk. Vitamin K intake was linked to ASCVD-related hospitalization with the highest intake of vitamin K1 and K2 related to 21% and 14% risk reduction respectively. They reported that the relationship between vitamin K1 intake and ASCVD was non-linear, with the benefits leveling off at 100 µg/day. Vitamin K2 intake was shown to have a U-shaped association with ASCVD hospitalization, suggesting that very high intakes might have adverse effects. Another prospective observational study by Dal Canto et al. (46) that investigated how vitamin D and K status related to cardiovascular health and mortality showed that low status of both micronutrients was related to higher left ventricular mass index, a marker of cardiac hypertrophy. Women with lower vitamin K had significantly lower left ventricular ejection fraction even while having normal vitamin D status.

Furthermore, Danziger et al. (47), analyzing data from 709 multiethnic adults from the Multi-Ethnic Study of Atherosclerosis (MESA) showed that low activities of VKDPs were associated with an increased risk of ischemic CVD. Among these proteins, the deficiency of MGP was linked to arterial stiffness and vascular calcification, and Gas6's inadequate activity is suggested to lead to atherosclerosis. Similarly, Hariri et al. (48) highlighted that lower intake of vitamin K2 leads to the accumulation of inactive MGP (dp-ucMGP) and is associated with increased arterial stiffness, vascular calcification, and heart failure. High dp-ucMGP levels were correlated with higher cardiovascular morbidity and mortality, increased pulse wave velocity (PWV), higher coronary artery calcification (CAC) scores, and worsened cardiac function in heart failure patients.

A systematic review by Hartley et al. (2015) found no evidence that vitamin K supplementation improves cardiovascular risk factors (49). On the other hand, in the Rotterdam study, a prospective population-based cohort study by Geleijnse et al. (44) that followed 4,807 Dutch men and women aged 55+ for 7.2 years, higher intake of menaquinone (vitamin K2) was associated with a 41% lower coronary heart disease (CHD) risk and a 57% lower CHD mortality. The association was significant even after adjusting for cardiovascular risk factors. However, no association was observed between phylloquinone intake and CHD or aortic calcification. Contrary to Geleijnse et al.'s finding, the Nurses' Health Study, a prospective study including 72,874 female nurses, found that women in the highest quintile of phylloquinone intake had a 21% risk reduction of total CHD but the association was weakened when it was adjusted for dietary factors. They also found that a higher intake of phylloquinone-rich vegetables led to a 35% decrease in the risk of CHD. However, they observed no association between Phyloloquinone intake with total or ischemic stroke risk **(**50). Additionally, a cross-sectional study on 60 chronic stroke survivors found that 82% of participants consumed below the Dietary Reference Intake (DRI) for vitamin K, suggesting that low vitamin K intake is prevalent in this high-risk population **(**51)**.**

On the other hand, Shea et al. (122) in a prospective cohort study from the Chronic Renal Insufficiency Cohort (CRIC) observed that vitamin K levels did not significantly impact atherosclerotic CVD risk (myocardial infarction, stroke, or peripheral artery disease). In clinical trials, vitamin K supplementation, while it improved vitamin K status, its role in the prevention of CVD progression hasn't been consistent (52). No significant impact was noted on carotid intima-media thickness (CIMT), arterial calcification, atherosclerosis, or arterial stiffness**.** Further randomized trials are needed to confirm the cardiovascular benefits of vitamin K, although observational studies have been promising.

Observational studies consistently demonstrate an inverse association between vitamin K2 intake and the risk of cardiovascular disease (CVD), arterial calcification, and metabolic disorders such as type 2 diabetes. These studies benefit from large sample sizes and long-term follow-up but are inherently limited by confounding factors (e.g., overall diet quality, lifestyle variables) and the inability to establish causality (44).

Conversely, randomized controlled trials (RCTs), while methodologically superior in reducing bias, have yielded more inconsistent results. Several RCTs investigating vitamin K supplementation, particularly with K1 or low-dose K2 (e.g., MK-4), have failed to show significant reductions in vascular calcification or improvement in insulin sensitivity. These null results may be explained by factors such as:

- **Short intervention duration**, insufficient to capture changes in slow-developing conditions like atherosclerosis.
- **Heterogeneity in baseline vitamin K status**, where participants with adequate levels may not benefit further.
- **Lack of stratification**, particularly by age, sex, baseline vascular health, or comorbidities.
- **Use of surrogate endpoints**, such as biomarkers, instead of clinical events (e.g., myocardial infarction or stroke) (53).

To address these gaps, we now emphasize the need for future RCTs to implement more rigorous participant stratification by baseline vitamin K status, sex, age, and disease risk profile, and to utilize higher-dose and longer-duration interventions with menaquinones, particularly MK-7, which demonstrate superior bioavailability and extrahepatic tissue targeting (54). These revisions appear primarily in the Cardiovascular and Metabolic Health sections and aim to contextualize the current body of evidence more critically and constructively.

Observational and interventional studies have shown promising associations between vitamin K—particularly K2—and improvements in bone and cardiovascular health; however, optimal dosing strategies remain under- defined, especially for specific populations such as premenopausal women, whose hormonal and metabolic variability may influence vitamin K metabolism and tissue requirements (30). Current evidence suggests that dietary intake of vitamin K1 (∼90–120 µg/day) is generally adequate to support hepatic γ-carboxylation and coagulation functions. However, higher and more sustained levels of vitamin K2 (particularly menaquinone-7) appear necessary to support extrahepatic functions such as arterial calcification inhibition and bone mineralization. Emerging clinical trials and safety data indicate that doses of MK-7 ranging from 90 to 180 µg/day are both safe and effective for improving vascular and skeletal markers over periods of 12 months or more, particularly in postmenopausal women and older adults. In contrast, much higher doses of MK-4 (up to 45 mg/day) have been used in osteoporosis studies, primarily in Japanese populations, but these require further validation in Western populations and broader demographics (55). Given the variability in absorption, baseline status, and health outcomes, we emphasize the need for future long-term, stratified RCTs to establish clear dose-response curves across age groups, sexes, and health conditions. This is critical to inform evidence-based, population-specific supplementation policies for both K1 and K2 (123).

## 6 Vitamin K and cancer

Vitamin K shows anticancer effects through different mechanisms, including apoptosis, cell cycle arrest, inhibition of metastasis, and autophagy activation. Vitamin K, by the release of cytochrome c and by the activation of mitochondrial pathways, Fas/FasL signaling, and caspase, triggers programmed cell death (apoptosis) (56, 57). In addition, vitamin K disrupts cyclin-dependent kinases (CDKs) leading to cell cycle arrest and stopping the growth of tumors. Vitamin K2 has been found to cause autophagy, a process that breaks down cells and recycles damaged parts to survive (56). This has two effects, In early-stage cancer, it can suppress tumor growth by removing damaged components inside the cell, or it can facilitate cancer cell survival under high stress such as chemotherapy in aggressive cancers. Moreover, vitamin K interferes with transcription factors such as c-Myc and NF-κB leading to a reduced cancer cell proliferation (driven by c-Myc) and decreased resistance to treatment (driven by NF-κB). Furthermore, vitamin K inhibits metastasis by reducing epithelial-mesenchymal transition (EMT), a transformation that helps cancer cells become more mobile and invasive (57).

Studies on the role of vitamin K on breast cancer are conflicting and vary between different forms of vitamin K. *in vitro* and *in vivo* studies showed that vitamin K2 (menaquinone-4) and vitamin K3 (menadione) have anticancer effects against triple-negative breast cancer (TNBC) and Her2+ breast cancer cells (57). In TNBC cells, vitamin K2 induced autophagy-dependent cell death (58). Similarly, Kiely et al. (124) reported that vitamin K2 significantly slows down the growth and spread of triple-negative breast cancer (TNBC) and HER2+ breast cancer in a dose-dependent manner. Beaudin et al. (59) also showed that vitamin K2 suppressed cell proliferation and inhibited cell migration, suppressing tumor growth. On the contrary, a study by Nimptsch et al. (60) reported that while higher dietary vitamin K2 intake was associated with lower overall and lung cancer incidence and mortality, no significant association was observed with breast cancer (pre-menopausal and post-menopausal). In addition, a large prospective cohort that followed 51,662 postmenopausal women for a median follow-up of 13.6 years studied how vitamin K intake was linked to breast cancer risk and mortality and observed that women who consumed the most vitamin K2 had a 26% higher risk of developing breast cancer compared to those who consumed the least and the risk increased with intake (61). In addition, breast cancer-related mortality increased with the highest intake (71% increase in risk). The evidence on the role of vitamin K1 on breast cancer is also mixed. Miyazawa et al. (58) reported that vitamin K1 increased cell proliferation in TNBC cells promoting tumor progression. In contrast, Palmer et al. (62) found that vitamin K1 intake was associated with a 20% reduction in cancer-related mortality, but the association was only significant in current or former smokers, suggesting it may be particularly beneficial in reducing cancer mortality in smokers.

Some studies show a promising vitamin K effect in cervical cancer. In the Sanxi CIN study in China, analyzing data from 2,304 women, 237 cases with cervical intraepithelial neoplasia (CIN2+), a precancerous condition that can lead to cervical cancer, researchers found that vitamin K intake was associated with reduced CIN risk, suggesting a potential role in early cervical cancer prevention (63). *In vivo* and *in vitro* studies showed that vitamin K3 led to apoptosis in cervical cancer cells, particularly in HPV-16-positive cervical cancer cells by increasing reactive oxygen species (57). In animal models, vitamin K3 combined with ultraviolet A (UVA) was confirmed to inhibit tumor growth.

In ovarian cancer cells, vitamin K2 inhibited ovarian cancer cell growth by disturbing the TR3/Nur77 signaling pathway, which is involved in cell survival and death regulation. Also, vitamin K3 triggers apoptosis through mitochondrial pathway activation and caspase enzymes which break down cancer cells (57). Furthermore, Shibayama-Imazu et al. (64) explained that vitamin K2 leads to oxidative stress and apoptosis in ovarian cancer cells, primarily by generating superoxide (O_2_•−), a highly reactive compound that damages cancer cells. The activation of caspase-3 by cytochrome C and the disruption of mitochondrial membrane potential results in cell death in these conditions.

## 7 Vitamin K and kidney diseases

Vitamin K plays a protective role in kidney health, particularly in its involvement in the activation of VKDPs such as MGP, Osteocalcin, and Gas6. MGP is a vitamin K-dependent protein that inhibits vascular calcification. Histopathological analysis by Wei et al. (65) examined renal biopsies in chronic kidney disease (CKD) and found that in the site of renal calcification, both carboxylated and uncarboxylated MGP were found, suggesting that vitamin K deficiency led to inadequate MGP activation and hence renal calcification which potentially leads to kidney damage. The calcification of the glomerular capillary may lead to the impairment of filtration, reducing the estimated glomerular filtration rate (eGFR) over time. In addition, uncarboxylated osteocalcin was reported to be positively correlated with CKD progression and proteinuria although OC levels did not consistently reflect vitamin K status, and Gas6 levels were elevated in CKD and inversely correlated with eGFR showing potential involvement in CKD progression (66).

A prospective cohort study by Groothof et al. (67) as part of the Prevention of Renal and Vascular End-stage Disease (PREVEND) study in the Netherlands examined 3,969 to examine the relationship between vitamin K status and kidney function. They found that high levels of dephosphorylated uncarboxylated MGP (dp-ucMGP), which is an indicator of poor vitamin K status, was associated with lower eGFR and a higher CKD risk and microalbuminuria. However, after adjusting for baseline kidney function and age, the association was not statistically significant. This implied that vitamin K deficiency may be a reflection of renal impairment rather than an independent risk factor. A cross-sectional study by Wei et al. (65) including 1,166 Flemish and 714 South African participants, found that higher levels of dp-ucMGP were associated with lower eGFR, lower kidney function, higher probability of progression to a more advanced CKD stage. Corroborating these findings is a prospective cohort study by Wei et al. (68) that followed 1,009 Flemish adults for 8.9 years and found that higher levels of dp-ucMGP levels were associated with greater eGFR decline and increased CKD progression risk. Participants who had the highest dp-ucMGP levels were 3.49 times more likely to develop CKD, reinforcing the idea that inadequate vitamin K intake may lead to kidney function deterioration over time. For a 5-fold increase in dp-ucMGP, eGFR declined by 3.15 ml/min/1.73 m^2^. Higher dp-ucMGP also predicted microalbuminuria, with an odds ratio of 4.70. On the other hand, a cross-sectional analysis of 842 patients with CVD by Parker et al. (69) showed that reduced kidney function was associated with lower ucMGP. For every 10 ml/min/1.73 m^2^ decrease in eGFR, ucMGP levels were 79 nM lower (after adjusting for confounders like age, sex, race, and biochemical factors). The study proposed that the reduction of ucMGP is caused by the suppression of MGP production due to altered metabolism in impaired kidney function. The lowering of ucMGP could then lead to vascular calcification worsening kidney dysfunction, and vascular calcification may further reduce circulating ucMGP levels by binding them to calcium deposits in blood vessels.

Supporting the beneficial role of vitamin K and vitamin K supplementation in kidney health, a study by Sun et al. (70) found that, patients with higher vitamin K status or those receiving supplementation had a 28% lower mortality risk and a a significantly higher eGFR (by 9.87 ml/min/1.73 m^2^) suggesting the protective role of vitamin K intake against kidney function decline. The long-term effects of vitamin K antagonists (VKA), a class of medications that inhibit the action of vitamin K, on kidney function were investigated by Posch et al. (71) in a retrospective cohort study of 14,432 patients with atrial fibrillation (AF) and CKD stages 3–4. They found that in VKA users, there was a faster decline in eGFR compared to non-users. Among VKA users eGFR decline was 24% compared to 14% in non-users. The association remained significant even after adjusting for age, baseline eGFR, CHA₂DS₂-VASc score, and comorbidities. The findings supported the “VKA-renal calcification hypothesis” suggesting that VKA use may accelerate vascular calcification in the renal microvasculature, leading to the progression of CKD. The impact of kidney transplantation on vitamin K status was explored by Kremer et al. (72), which included two cohorts: one with 578 kidney transplant recipients and another with 124 patients undergoing kidney transplantation. They found that after kidney transplantation, dp-ucMGP levels declined by half, suggesting that elevated levels in CKD patients may be due to impaired renal clearance rather than deficiency. This raised concern about the use of dp-ucMGP as a vitamin K status marker in CKD patients as it may be reflective of kidney function rather than vitamin K insufficiency. This aligns with the findings of Voskamp et al. (73), which showed that VKA users had a slightly greater annual decline in eGFR, although the difference was not significant.

## 8 Vitamin K and brain diseases

Vitamin K plays a crucial role in the brain with evidence suggesting its involvement in cognitive health, neuroprotection, and neurodegenerative diseases. The neuroprotective effects of vitamin K are largely related to its role in sphingolipid metabolism and the activation of vitamin K-dependent proteins such as Gas6 and Protein S. MK-4 is involved in sphingolipid biosynthesis and metabolism, which is crucial for brain neuronal membranes and myelin sheath formation (74, 75). Alterations in sphingolipid metabolism are linked to neurodegenerative diseases, including Alzheimer's disease (AD) and Parkinson's disease (75). Vitamin K enhances the production of brain sulfatides, a class of sphingolipids necessary for myelination and neuronal function (76). Vitamin K deficiency and the use of vitamin K antagonists such as warfarin, reduce the synthesis of sulfatides affecting brain function (75). Vitamin K-dependent proteins such as Gas6 and Protein S play important roles in neuronal survival, anti-apoptotic signaling, and myelination (74). Gas6 activates TAM receptors (Tyro3, Axl, Mer) that regulate inflammation, cell survival, and myelination in the brain (77). It also protects hippocampal neurons from apoptosis and amyloid-beta-induced toxicity**,** which is relevant to Alzheimer's disease (75)**.** Protein S provides antithrombotic and neuroprotective effects and protects neurons from excitotoxic injury via the Tyro3-PI3K-Akt signaling pathway (75). In addition, vitamin K1 and MK-4 protect neurons and oligodendrocytes from oxidative stress and apoptosis (75, 78). Vitamin K protects developing oligodendrocytes, which are highly vulnerable to oxidative stress and play a role in white matter injury and neurodevelopmental disorders, including periventricular leukomalacia (PVL) and cerebral palsy, from oxidative damage (78). MK-4 helps suppress microglial inflammation responses which if overactivated drives neuroinflammation and contributes to neurodegenerative diseases like Alzheimer's disease (79). MK-4 dose-dependently suppressed the production of proinflammatory cytokines (IL-1β, TNF-α, and IL-6) in LPS-stimulated MG6 mouse microglial cells. It blocks key inflammation signals (NF-κB and TNF-α pathways), by preventing phosphorylation of NF-κB p65, a critical step in NF-κB activation, thereby reducing inflammation.

A longitudinal study by Booth et al. (80) based on the Rush Memory and Aging Project (MAP) demonstrated that higher MK-4 in the brain was linked to better cognitive function and 17%–20% lower odds of dementia or mild cognitive impairment, fewer signs of Alzheimer's disease, Parkinson's disease and other forms of neurodegeneration. Higher plasma phylloquinone levels (vitamin K1) were linked to better cognitive function and a slower decline rate. Similarly, a study by Chouet et al. (74) from the CLIP study found that higher vitamin K1 intake from the diet was associated with better cognitive performance, as measured by the Mini-Mental State Examination (MMSE), suggesting protection against cognitive deterioration. Reinforcing these findings, a study by Kiely et al. (81) from the ELDERMET cohort showed that higher vitamin K intake is linked to better cognitive function measured by Mini-Mental State Examination (MMSE). Subjects with the highest vitamin K intake (>121 µg/day) had significantly better cognition than those in the lowest intake group (<73 µg/day). Furthermore, dietary and serum phylloquinone were inversely correlated with IL-6, a key pro-inflammatory cytokine linked to cognitive impairment. Further supporting this, Presse et al. (82) found that participants with higher serum phylloquinone levels performed better on the verbal episodic memory task, particularly in free recall tasks, but had no significant impact on executive functions or processing speed. The relationship between serum phylloquinone and memory performance followed a logarithmic pattern, with improvements in memory scores plateauing beyond a certain level, suggesting that beyond this level, additional vitamin K may not significantly enhance cognition. A study by Tamadon-Nejad et al. (83) demonstrated that warfarin-induced vitamin K deficiency in rats led to cognitive deficits, particularly in spatial learning, as assessed by the Morris water maze. These rats exhibited altered sphingolipid metabolism, reduced MK-4 levels, and impaired exploratory behavior, reinforcing the essential role of vitamin K in cognitive function. Moreover, higher vitamin K intake was related to better behavioral performance, assessed by the frontotemporal behavioral rating scale (FBRS), which evaluates self-control, physical neglect, mood disorders, and loss of interest. Lower vitamin K intake was linked to higher FBRS scores (74).

## 9 Vitamin K, insulin sensitivity, and diabetes

Vitamin K has been shown to have a role in glucose metabolism, insulin sensitivity, and T2DM. It is suggested that Vitamin K may enhance insulin sensitivity through the carboxylation of osteocalcin, a non-collagenous protein that plays key roles in energy metabolism and glucose regulation (84–86). Vitamin K (particularly menaquinone-4, MK-4) may enhance glucose-stimulated insulin secretion, acting as an incretin-like molecule (85). Ho et al. (87) reported that Mk-4 increases cyclic adenosine monophosphate and enhances glucose-stimulated insulin secretion in β-cells without causing hypoglycemia (a risk with traditional insulin secretagogues). Additionally, the activation of adiponectin helps to improve insulin sensitivity in muscle and liver tissues (88). This theory, however, was challenged by studies that showed glycemic improvements were independent of an increase in serum adiponectin levels (89, 90). Although adiponectin was shown to increase with vitamin K1 supplementation, it did not mediate glycemic improvements (89, 90). Moreover, vitamin K modulates AMP-activated protein kinase (AMPK) and Sirtuin 1 (SIRT1) pathways, which enhance glucose uptake, mitochondrial function, and lipid metabolism (88). Vitamin K also plays a role in the modulation of inflammation and oxidative stress, which may improve glucose homeostasis (89, 90). It reduces oxidative stress markers such as NF-κB, ROS (O2−, OH), and aldose reductase (AR) and increases antioxidant enzyme activities such as Superoxide Dismutase (SOD), Glutathione (GSH) and Catalase (CAT) which protect against diabetic related complications such as diabetic nephropathy, neuropathy, and vascular complications (88).

A systematic review by Karamzad et al. (88) reported the role of vitamin K in glycemic control, including a reduction in blood glucose level, increase in fasting serum insulin, reduction in HbA1c, improvements in HOMA-IR and β-cell function, and improvements in post-OGTT glucose and insulin levels. Additionally, a cross-sectional study by Yoshida et al. (91) found that a higher intake of phylloquinone was associated with greater insulin sensitivity and better glycemic control. Individuals with the highest quantile of phylloquinone intake had significantly lower 2-hour post-OGTT insulin levels (72.7 μU/ml) compared to those in the lowest quintile (81.0 μU/ml). Glucose levels 2-h post-OGTT were also lower in individuals with higher intake (101.9 mg/dl) compared to those with lower intake (106.3 mg/dl). However, the study found no significant association between phylloquinone intake and fasting insulin, fasting glucose, HOMA-IR or HbA1c. Similarly, in a four-week double-blind RCT in 82 prediabetic premenopausal women, Rasekhi et al. (89, 90) found that a 1,000 µg/day supplementation of phylloquinone significantly improved 2-h post-OGTT glucose and insulin levels but there was no significant change in fasting blood glucose or fasting insulin (89, 90). Similarly, a meta-analysis study by Suksomboon et al. (92) pooled data from 8 RCTs with 1,077 participants and vitamin K supplementation did not significantly affect insulin resistance as measured by HOMA-IR, fasting plasma glucose, and fasting insulin. The lack of effect could be attributed to factors like baseline insulin sensitivity as many of the trials involved older adults and post-menopausal women, a population with naturally declining insulin sensitivity. Biomarkers such as total osteocalcin, leptin levels, HOMA-IR, and insulin sensitivity index did not change with vitamin K1 supplementation (89, 90).

Beulens et al. (84) analyzed data from the European Perspective Investigation into Cancer and Nutrition (EPIC)-NL study and found that T2DM risk lowered with the intake of vitamin K1 (exhibiting non-linear inverse association) and vitamin K2 (exhibiting linear inverse association) (HR = 0.81, 95% CI: 0.66–0.99 for phylloquinone; HR = 0.93 per 10 µg increment for menaquinones). Corroborating this, a longitudinal study by Ibarrola-Jurado et al. (86) that followed 1,925 elderly individuals at high cardiovascular risk found that higher intake of phylloquinone lowered the risk of T2DM over a 5.5-year follow-up. An increase of 100 μg/day in intake led to a 17% reduction in T2DM risk (HR = 0.83, 95% CI: 0.712–0.967, *P* = 0.017) (86). Moreover, according to data from three major cohorts (EPIC-InterAct Study, Diabetes Genetics Replication and Meta-analysis Consortium and UK biobank) which included a total of 69,647 individuals with T2DM, each natural log increase in circulating phylloquinone was associated with a 7% T2DM risk reduction (RR = 0.93, 95% CI: 0.89–0.97) (93). Vitamin K also has a protective role against diabetes-related complications such as diabetic nephropathy, diabetic neuropathy, and comorbidities associated with diabetes (85, 88).

## 10 Vitamin K and vascular calcification

Vascular calcification (VC) is a complex**,** active pathological process regulated by multiple factors, including uremic toxins, oxidative stress, inflammation, and disturbed calcium-phosphate metabolism (94). Inflammation is a major driver of VC, as macrophages promote osteoblastic differentiation of vascular smooth muscle cells (VSMCs). Inflammatory cytokines such as Interleukin-1β (IL-1β), Interleukin-6 (IL-6), Tumor Necrosis Factor-α (TNF-α) and Oncostatin M (OSM) activate the NF-κB signaling pathway, which leads to VSMC transformation into osteoblast-like cells that deposit calcium (95). Vitamin K inhibits the NF-κB signaling, thereby suppressing inflammation. In addition, VKDP regulates vascular calcification, with MGP being the most critical inhibitor of calcification (96). Active MGP prevents VSMC transformation into osteoblast-like cells, reducing calcium deposition. It inhibits bone morphogenetic protein-2 (BMP-2), a driver of vascular mineralization (94). Other VKDPs such as Gas6, prevent VSMCs from apoptosis and lead to the reduction of VC. Protein S reduces VSMC calcification through TAM receptor signaling (95).

In animal studies, vitamin K supplementation has been shown to prevent and even reverse VC (94). Rodent models of CKD consistently showed that warfarin accelerates VC. High-dose vitamin K supplementation reverses warfarin-induced arterial calcification in rats (97). In observational studies, phylloquinone (K1) supplementation (500 µg/day) slowed CAC progression in community-dwelling adults (97). A cohort study by Dai et al. (98) found that patients with higher dp-ucMGP had significantly higher scores of CAC and aortic valve calcium (AVC). However, when adjusted for confounding variables, dp-ucMGP was not identified as an independent determinant of vascular calcification, suggesting that vitamin K deficiency is not directly involved in vascular calcification development (98). Randomized controlled trials (RCTs) have yielded mixed findings on the role of vitamin K in vascular calcification. The VitaVask trial showed that Vitamin K1 supplementation reduced aortic calcification by 56% and coronary calcification by 68% (99). Analysis of the Agatston score showed a significant reduction in CAC progression at 18 months (*p* = 0.028) (99). In contrast, studies evaluating vitamin K supplementation have not consistently shown similar benefits. For example, MK-7 supplementation (90 µg/day) was found to slow carotid artery calcification in CKD patients, whereas MK-4 (45 mg/day) had no significant effect on CAC progression in diabetic patients (95). A randomized controlled trial involving 40 vitamin K-deficient kidney transplant recipients found that vitamin K supplementation did not change serum calcification propensity, suggesting it may not directly influence systemic calcification processes in the short term (100). Furthermore, the Trevasc-HDK trial involving 178 hemodialysis patients found that vitamin K2 supplementation did not have a significant effect on coronary artery calcification score, aortic valve calcium score, and arterial stiffness (101). This suggests that vitamin K2 may not be effective in reversing existing calcification. Similarly, the iPACK-HD study found that the supplementation of Vitamin K1 in end-stage kidney disease patients on hemodialysis had no significant effect on coronary artery calcification over 12 months (102). In the RenaKvit trial, vitamin K supplementation did not reduce CAC scores and AAC progression in dialysis patients (20). Imaging markers of vascular calcification fail to show that vitamin K supplementation can reduce existing vascular calcifications. No improvement was observed in coronary artery Agatston, aortic Agatston, and valve calcification scores with vitamin K supplementation (103).

A systematic review by Li et al. (104) concluded that vitamin K supplementation slows CAC progression but does not consistently impact other vascular or valvular calcifications**.** Similarly, a review by Neofytou et al. (96), analyzing data from multiple trials (Trevasc-HDK, Valkyrie, and ViKTORIES), found no significant reduction in CAC, aortic calcification, or vascular stiffness with vitamin K supplementation**.** Likewise, Roumeliotis et al. (105) reported that MK-7 supplementation had no significant effect on VC or arterial stiffness in kidney patients**.** Studies have consistently shown that warfarin users exhibit greater vascular calcification compared to non-users**.** A meta-analysis by Kosciuszek et al. (106) found that vitamin K antagonist use was associated with increased CAC, extra-coronary vascular calcification, and a significant increase in aortic valve calcification. Similarly, Levy et al. (107) reported that lower vitamin K status is associated with greater CAC in CKD patients, and warfarin use accelerates VC by preventing MGP activation. Patients on warfarin showed higher CAC and iliac artery calcification compared to non-users (97). Contrary to previous findings, an RCT conducted by Zwakenberg et al. (93, 108) found that MK-7 supplementation (360 µg/day) tended to increase active vascular calcification compared to placebo. Although the association was not statistically significant, it showed a potential increase in vascular calcification activity. The MK-7 group had higher initial CAC scores that might have predisposed them to greater progression. No significant effect was observed on CAC mass measured via computed tomography (CT) (93, 108). Given the conflicting evidence, the role of vitamin K in vascular calcification remains unclear. Most of the research on the role of vitamin K in VC has been conducted in kidney disease patients, where pre-existing VC may have influenced the findings**.** Future studies should focus on determining optimal doses, assessing long-term efficacy, and comparing the differential effects of vitamin K1 and K2 supplementation**.**

Observational studies have generally reported an inverse association between vitamin K2 intake, particularly long-chain menaquinones such as MK-7, and the extent or progression of vascular calcification. These findings are especially evident in cohorts at increased cardiovascular risk, including elderly populations and patients with chronic kidney disease (CKD). However, randomized controlled trials (RCTs) evaluating vitamin K supplementation have produced more variable results, with several failing to demonstrate statistically significant changes in calcification progression (109).

The observed discrepancies likely stem from multiple factors. First, the form of vitamin K used is a major determinant of efficacy: K2 (especially MK-7) is more bioavailable and has a longer half-life than K1, allowing better extrahepatic tissue targeting. Second, study populations differ markedly across trials; individuals with CKD or advanced calcification may have reduced vitamin K metabolism, altered absorption, or more irreversible vascular pathology, potentially limiting the impact of supplementation. Third, variations in study design, such as intervention duration, vitamin K dose, baseline vitamin K status, and the methods used to quantify calcification (e.g., coronary artery calcium scoring vs. aortic imaging)—contribute to the inconsistent findings (110, 111).

Given the current state of evidence, we propose that vitamin K2—particularly MK-7—holds promise for reducing or slowing vascular calcification, especially in populations with subclinical deficiency or elevated cardiovascular risk. However, its routine clinical use for this indication cannot yet be universally recommended. Larger, well-powered, long-term RCTs with standardized endpoints and stratified participant selection are needed to confirm the clinical efficacy of vitamin K2 in vascular calcification prevention and management (112).

## 11 Role of vitamin K during pregnancy

Vitamin K has a crucial role in maternal and neonatal health, mainly due to its involvement in blood coagulation. Maternal vitamin K levels can significantly influence neonatal health. A systematic review by Shahrook et al. (113) reported that vitamin K supplementation increased maternal plasma vitamin K1 levels significantly, leading to improved neonatal cord serum vitamin K1 levels and higher vitamin K content in maternal breast milk. However, no significant effect was found for reducing neonatal bleeding. Preterm infants are at an increased risk of periventricular hemorrhage (PVH) due to immature coagulation systems. A systematic review by Crowther & Crosby (114) evaluated the effect of antenatal vitamin K administration in women at risk of preterm birth. The analysis included data from seven trials, including 843 women. They found that prenatal vitamin K administration led to no significant reduction in all grades of PVH. However, there was a significant reduction in severe PVH (grade 3 and 4) in some studies (114). Despite this, when lower-quality trials were excluded, the protective effect was no longer statistically significant, indicating the need for higher-quality randomized controlled trials (RCTs) to confirm these findings. Furthermore, the review assessed long-term neurodevelopmental outcomes and found no significant differences in cognitive or motor development between infants who received vitamin K exposure *in utero* and those who did not (114). A randomized controlled trial by Liu et al. (115) investigated the effects of maternal antenatal administration of vitamin K1 on neonatal coagulation and the incidence of periventricular-intraventricular hemorrhage (PIVH) in premature infants (<35 weeks gestation). Their findings highlighted those preterm infants had significantly lower levels of vitamin K-dependent coagulation factors (II, VII, IX, and X) compared to full-term infants. Maternal antenatal vitamin K1 supplementation significantly improved the levels of factors II, VII, and X, reducing both the overall incidence and severity of PIVH (115). Specifically, the study found that PIVH incidence was lower in the vitamin K1 group (32.5%) compared to the control group (54.0%), with a significant reduction in severe PIVH cases (5.0% vs. 20.0%) (115). These results suggest that maternal vitamin K1 supplementation may play a protective role against neonatal hemorrhagic complications in high-risk pregnancies. There are not many up-to-date RCTs investigating the role of vitamin K in pregnancy. The existing studies exhibit inconsistency and warrant further clinical investigation to understand the potential benefits of vitamin K interventions during pregnancy.

## 12 Summary

Table 1 summarizes key studies examining the effects of vitamin K on various health outcomes across different population groups. Most studies targeted postmenopausal women or older adults, focusing on bone and cardiovascular health. Several randomized controlled trials (RCTs) demonstrated positive effects of vitamin K2 supplementation, particularly MK-4 and MK-7, on bone mineral density (116) and arterial elasticity (53). Additionally, K2 improved insulin sensitivity in women with polycystic ovary syndrome (PCOS) (89, 90). A large cohort study found that higher dietary intake of vitamin K2, but not K1, was associated with reduced coronary heart disease risk (117). However, not all findings were favorable. Shea et al. (40, 41) reported no effect of vitamin K1 on cognitive decline in elderly participants. Overall, the evidence highlights the potential of vitamin K, especially K2, in supporting bone and cardiovascular health, though effects may vary by form, dose, and target outcome.

**Table 1:** Summary of key studies on vitamin K and health outcomes by population group.

Study (Author, Year)	Population	Age range	Study design	Vitamin K type	Dose	Duration	Main outcome
Booth et al. (120)	Postmenopausal women	55–70	Observational (Cross-sectional)	K1	Dietary intake (median 122 µg/day)	—	Lower bone loss rates with higher K1 intake
Braam et al. (116)	Healthy postmenopausal women	50–60	RCT	K2 (MK-4)	45 mg/day	3 years	Improved bone mineral density
Beulens et al. (117)	Adult women and men	20–70	Prospective cohort	K1 and K2	Dietary intake	8.1 years	K2 associated with reduced coronary heart disease risk
Knapen et al. (53)	Postmenopausal women	55–65	RCT	K2 (MK-7)	180 µg/day	3 years	Increased arterial elasticity
Rasekhi et al. (89, 90)	Women with PCOS	18–40	RCT	K2 (MK-7)	180 µg/day	8 weeks	Improved insulin sensitivity
Shea et al. (40, 41)	Elderly men and women	≥65	RCT	K1	500 µg/day	3 years	No effect on cognitive decline

RCT, Randomized Controlled Trial; PCOS, Polycystic Ovary Syndrome; MK-4/MK-7, Menaquinone-4/Menaquinone-7 (forms of Vitamin K2).

**Figure 1:**
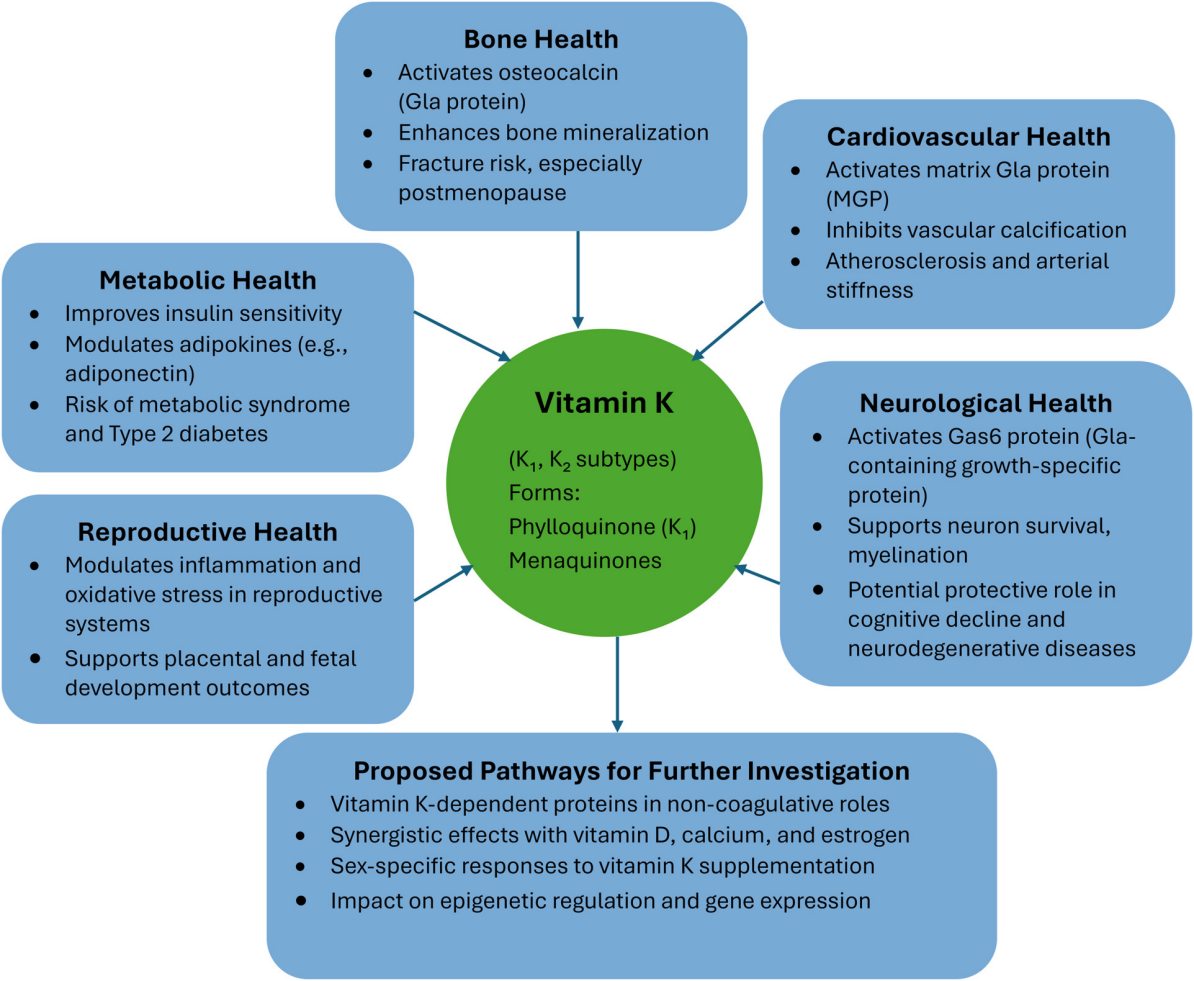
Vitamin K and health effects.

Figure 1 provides an overview of the diverse physiological roles and health benefits of vitamin K, encompassing both K₁ and K₂ subtypes. Vitamin K contributes significantly to bone health by activating osteocalcin and enhancing bone mineralization, thereby reducing fracture risk, particularly in postmenopausal women. In cardiovascular health, it activates matrix Gla protein (MGP), which inhibits vascular calcification and reduces arterial stiffness and atherosclerosis. The vitamin also plays a role in neurological health by activating Gas6 protein, supporting neuron survival and myelination, and potentially protecting against cognitive decline and neurodegenerative diseases. In the area of metabolic health, vitamin K improves insulin sensitivity, modulates adipokines like adiponectin, and may lower the risk of metabolic syndrome and type 2 diabetes. Regarding reproductive health, it modulates inflammation and oxidative stress in reproductive tissues and supports placental and fetal development. The figure also suggests proposed pathways for further investigation, including exploring vitamin K-dependent proteins in non-coagulative roles, its synergy with vitamin D, calcium, and estrogen, sex-specific responses to supplementation, and its effects on epigenetic regulation and gene expression.

**Figure 2:**
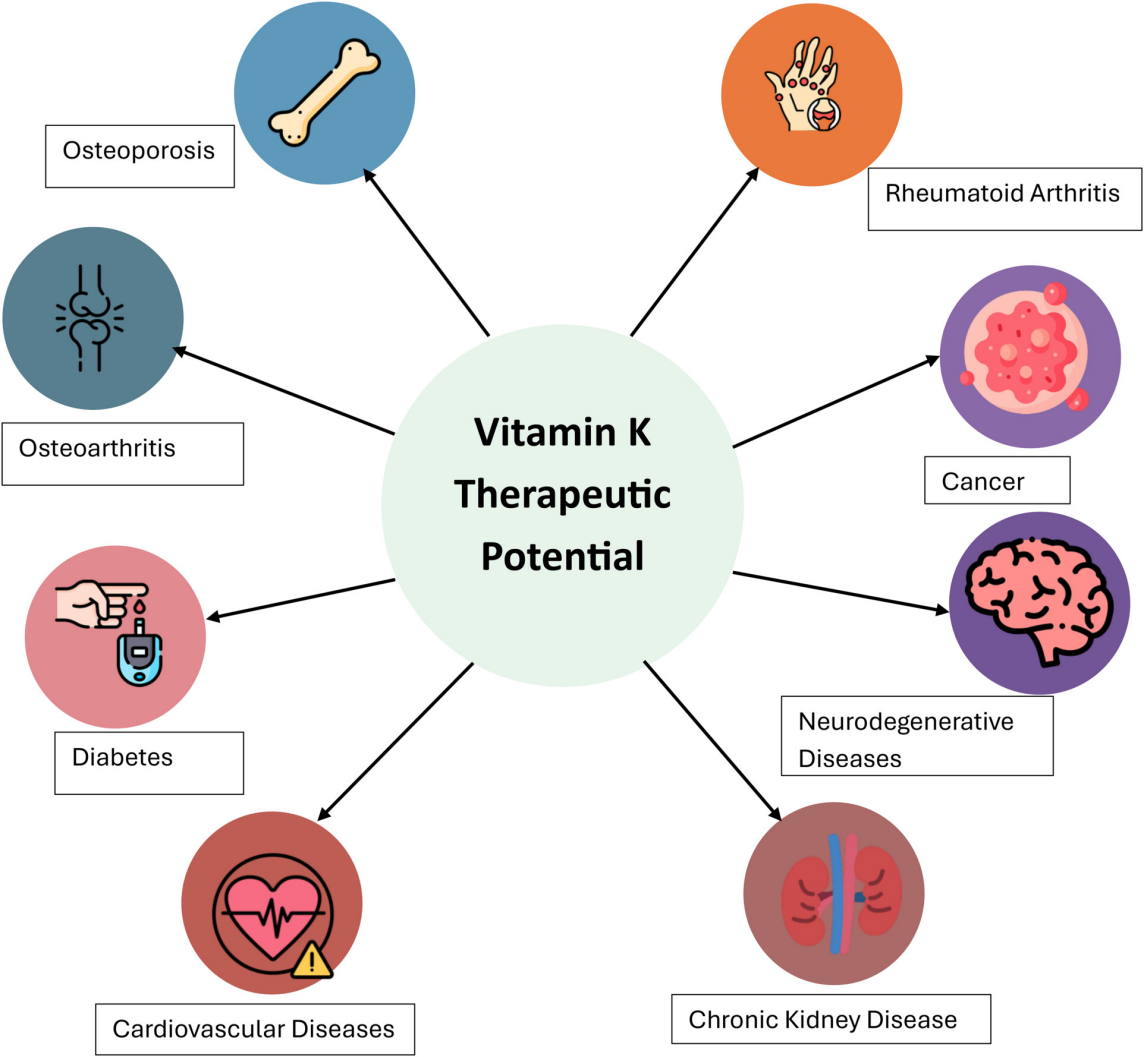
The therapeutic potential of VK2 in various disease states (119).

Figure 2 illustrates the broad therapeutic potential of Vitamin K2 (VK2) across a range of chronic and degenerative diseases. Positioned centrally, VK2 is shown to potentially impact eight major health conditions: osteoporosis, osteoarthritis, rheumatoid arthritis, cardiovascular disease, chronic kidney disease, cancer, neurodegenerative disease, and diabetes. Each condition is linked to VK2 via directional arrows, suggesting its beneficial role in either treatment or prevention. The diagram highlights VK2 as a multifaceted nutrient with emerging significance in clinical and preventative health strategies beyond its traditional roles in bone and blood health.

## 13 Conclusion

Vitamin K is a multifunctional nutrient with several functions, including bone health, cardiovascular health, insulin sensitivity, cancer prevention, immune regulation, brain health, and maternal and child health. While some studies report vitamin K as having positive health outcomes, others report no significant change in certain health conditions. In bone health, vitamin K is suggested to have a role in the preservation of bone mass and strength, but findings from clinical trials are inconclusive. Similarly, while observational studies associate vitamin K with positive cardiovascular outcomes, randomized controlled trials showed inconsistent findings, particularly after adjusting for confounders. Studies on metabolic health suggest that vitamin K may lead to improvement in glucose metabolism and β-cell function, but interventional studies have not shown a significant effect on insulin resistance, especially in those with pre-existing insulin resistance. In addition, while *in vitro* studies support the anti-cancer effects of vitamin K, epidemiological studies are inconsistent, some suggesting a protective effect and others raising concerns about increased risk in certain populations. Neuroprotective benefits of vitamin K have been observed in some studies associating higher intake with improved cognitive function and reduced neurodegenerative disease risk, however, clinical trials are lacking. In kidney disease, Vitamin K deficiency has been reported to lead to CKD progression, lower estimated eGFR, and increased vascular calcification, yet whether this deficiency is a cause, or a consequence remains unclear. In maternal and fetal health, limited research exists on optimal maternal vitamin K intake and its effects on neonatal outcomes. In a few of the studies done on the role of vitamin K in pregnancy, vitamin K has been reported to play crucial roles in neonatal coagulation with some studies reporting antenatal supplementation to reduce the risk of severe periventricular hemorrhage (PVH) in preterm infants. However, there is no consensus established on routine maternal supplementation and long-term effects remain unclear. Even though Vitamin K's importance in physiological processes is recognized, there is a significant gap in the literature in understanding its role in women's health. The existing studies are limited by small sample sizes, short study durations, and an overrepresentation of mixed-gender or male-dominated cohorts. Many studies are done on populations with pre-existing health conditions, such as diabetes or CKD, limiting generalizability to healthy individuals. Women are underrepresented in certain clinical trials, particularly in bone, cardiovascular, metabolic, and reproductive health research, leaving gaps in knowledge on vitamin K's role in female-specific health outcomes. Some studies have found a distinct physiological role between different forms of vitamin K (K1 vs. K2) but the differences and mechanisms involved are not clearly understood. Additionally, many studies measure vitamin K at a single point in time, failing to capture changes over time and long-term impact.

## 14 Recommendations

Based on the gaps identified, further research should address existing gaps and limitations in the literature by exploring several critical aspects of vitamin K's role in women's health. Clinical trials on the female population should be conducted to address the underrepresentation of the female demographics in many of the studies identified in this review for better generalizability. Diverse female populations should be sampled, including premenopausal and postmenopausal women, individuals with varying baseline vitamin K intake levels, and those from different ethnic and genetic backgrounds to investigate impact across varying populations. Besides, optimizing dosing strategies and improving bioavailability are essential to enhance its therapeutic effectiveness. To assess the efficacy of vitamin K in BMD preservation and fracture risk reduction, long-term studies should be conducted. Given the promising results from *in vitro* studies, further clinical trials should examine its potential anti-cancer properties and the distinct effects against specific cancer types among the different vitamin K forms. The role of vitamin K supplementation on vascular calcification should be studied by more randomized controlled trials and longitudinal studies, particularly in healthy population as many of the current studies focused on kidney disease patients. Future studies should also include younger and premenopausal women to clarify vitamin K's role in glucose metabolism, insulin sensitivity, and type 2 diabetes prevention. Additionally, well-controlled clinical trials are critically needed to evaluate the impact of maternal vitamin K intake on pregnancy outcomes, neonatal health, and long-term child development. Studies should also explore the different effects of vitamin K forms (K1 vs. K2) providing clearer insights into their respective roles.
