# Correction: Vitamin K and women’s health: a review

**DOI:** 10.3389/fgwh.2025.1695134

**Published:** 2025-11-04

**Authors:** Sharifa AlBlooshi

**Affiliations:** Department of Health Sciences, College of Natural and Health Sciences, Zayed University, Dubai, United Arab Emirates

**Keywords:** vitamin K, women's health, bone health, cardiovascular disease, diabetes, menopause, immune system, cancer

**Abstract**

Adding/removing text

In the abstract, the term was incorrectly written with a lowercase abbreviation as: “**matrix gla protein**”

This has been corrected to read: “**matrix Gla protein**”

The original version of this article has been updated.

In the abstract, the sentence was incorrectly written without a comma after “**Similarly” and with “diseases**” in plural form as: “**Similarly in kidney diseases, vitamin K has been linked to chronic kidney disease progression and vascular calcification**.”

This has been corrected to read: “**Similarly, in kidney disease, vitamin K has been linked to chronic kidney disease progression and vascular calcification**.”

The original version of this article has been updated.

**Error in figure/table**

Wrong content

There was a mistake in table 1 as published. The entry included:

“Iwamoto et al. (118) Osteoporotic women 60–75 RCT K2 (MK-4) 45 mg/day 2 years Decreased fracture incidence.” This study has been removed.

The corrected Table 1 appears below.

**Table:** 

Study (Author, Year)	Population	Age range	Study design	Vitamin K type	Dose	Duration	Main outcome
Booth et al. (120)	Postmenopausal women	55–70	Observational (Cross-sectional)	K1	Dietary intake (median 122 µg/day)		Lower bone loss rates with higher K1 intake
Braam et al. (116)	Healthy postmenopausal women	50–60	RCT	K2 (MK-4)	45 mg/day	3 years	Improved bone mineral density
Beulens et al. (117)	Adult women and men	20–70	Prospective cohort	K1 and K2	Dietary intake	8.1 years	K2 associated with reduced coronary heart disease risk
Knapen et al. (53)	Postmenopausal women	55–65	RCT	K2 (MK-7)	180 µg/day	3 years	Increased arterial elasticity
Rasekhi et al. (89, 90)	Women with PCOS	18–40	RCT	K2 (MK-7)	180 µg/day	8 weeks	Improved insulin sensitivity
Shea et al. (40, 41)	Elderly men and women	≥65	RCT	K1	500 µg/day	3 years	No effect on cognitive decline

RCT, randomized controlled trial; PCOS, polycystic ovary syndrome; MK-4/MK-7, Menaquinone-4/Menaquinone-7 (forms of vitamin K2).

The original version of this article has been updated.

**Text Correction**

The text was incorrectly written with a lower case as: “**brussels sprouts**”

A correction has been made to the section [Introduction, page 2, paragraph 2]:

“**Brussels sprouts”**

The original version of this article has been updated.

The text was incorrectly written as: “…the European Communities **recommends** 75 µg/day (4).”

A correction has been made to the section [Introduction, page 2, paragraph 3]:

“…the European Communities **recommend** 75 µg/day (4).”

The original version of this article has been updated.

The text was incorrectly written as: “In addition to acting as an enzyme cofactor, vitamin K plays

roles such as having **anti-inflammation** effects…”

A correction has been made to the section [Introduction, page 2, paragraph 4]:

“In addition to acting as an enzyme cofactor, vitamin K plays roles such as **anti-inflammatory** effects…”

The original version of this article has been updated.

The text was incorrectly written as: “…exhibits an antioxidative activity 10–100 times **higher than other** antioxidants …”

A correction has been made to the section [Introduction, page 2, paragraph 4]:

“…exhibits antioxidative activity 10–100 times **higher than that** of other antioxidants …”

The original version of this article has been updated.

The text was incorrectly written as: “…protective **effect** on neurons (4).”

A correction has been made to the section [Introduction, page 2, paragraph 6]:

“…protective **effects** on neurons (4).”

The original version of this article has been updated.

The sentence ended incompletely as: “…which influence bone health, cardiovascular health, immune function, and **other**.”

A correction has been made to the section [Introduction, page 2, paragraph 6]:

“…which influence bone health, cardiovascular health, immune function, and **other health outcomes**.”

The original version of this article has been updated.

The heading was incorrectly formatted as inconsistent with other section headings:

“**1.1 The mechanisms of vitamin K1 and K2**”

A correction has been made to the section [Section 1.1, page 2, Heading]:

“**1.1 The mechanisms of vitamin K1 and K2**”

The heading is now formatted consistently with other section headings (red font and bold).

The original version of this article has been updated.

The heading was incorrectly written as:

“**2 Vitamin K with osteoporosis and bone health**”

A correction has been made to the section [Section 2, page 2, Heading]:

“**2 Vitamin K, osteoporosis, and bone health**”

The original version of this article has been updated.

The text was incorrectly written with a minor grammatical mistake as: “This highlights **the** two forms of vitamin K might have different roles …”

A correction has been made to the section [Section 2, page 3, paragraph 3]:

“This highlights **that the** two forms of vitamin K might have different roles …”

The original version of this article has been updated.

The heading was incorrectly written as:

“**4 Vitamin K and immunity and the immune system”**

A correction has been made to the section [Section 4, page 4, Heading]:

“**4 Vitamin K and immunity”**

The original version of this article has been updated.

The text was incorrectly written as: “…higher plasma phylloquinone **(vitamin K1)** were inversely associated with inflammatory markers …”

A correction has been made to the section [Section 4, page 4, paragraph 3]:

“higher plasma phylloquinone **(vitamin K1) concentrations** were inversely associated with inflammatory markers …”

The original version of this article has been updated.

The text was incorrectly written as: “…**less intake** of vitamin K2 leads to…”

A correction has been made to the section [Section 5, page 5, paragraph 2]:

“…**lower intake** of vitamin K2 leads to …”

The original version of this article has been updated.

The text was incorrectly written as:

“In the Framingham Offspring Study, Higher vitamin K intake was associated with lower CVD risk (49). In the Rotterdam study, a prospective population-based cohort study by Geleijnse et al. (44) that followed 4,807 Dutch men and women aged 55+ for 7.2 years, higher intake of menaquinone (vitamin K2) was associated with a 41% lower coronary heart disease (CHD) risk and a 57% lower CHD mortality.”

A correction has been made to the section [Section 5, page 5, paragraph 3]:

“A systematic review by Hartley et al. (2015) found no evidence that vitamin K supplementation improves cardiovascular risk factors (49). On the other hand, in the Rotterdam study, a prospective population-based cohort study by Geleijnse et al. (44) that followed 4,807 Dutch men and women aged 55+ for 7.2 years, higher intake of menaquinone (vitamin K2) was associated with a 41% lower coronary heart disease (CHD) risk and a 57% lower CHD mortality.”

The original version of this article has been updated.

The text was incorrectly written with minor grammatical mistake and incorrect capitalization as: “**However, no association was observed with phylloquinone intake with CHD or aortic calcification.** Contrary to Geleijnse et al.'s finding, The Nurses’ Health Study, …”

A correction has been made to the section [Section 5, page 5, paragraph 3]:

“**However, no association was observed between phylloquinone intake and CHD or aortic calcification**. Contrary to Geleijnse et al.'s finding, the Nurses’ Health Study, …”

The original version of this article has been updated.

The text was incorrectly written with typographical errors as: “…**and improvements in bone and cardiovascular health, optimal dosing strategies remain underdefined,**..”

A correction has been made to the section [Section 5, page 6, paragraph 8]:

“**… and improvements in bone and cardiovascular health; however, optimal dosing strategies remain under-defined,** …”

The original version of this article has been updated.

The text was incorrectly written as: “… **for a median year of 13.6 years**…”

A correction has been made to the section [Section 6, page 6, paragraph 2]:

“… **for a median follow-up of 13.6 years** …”

The original version of this article has been updated.

The text was incorrectly written as: “… **compared to who consumed the least** …”

A correction has been made to the section [Section 6, page 6, paragraph 2]:

“… **compared to those who consumed the least** …”

The original version of this article has been updated.

The text was incorrectly written with unit formatting errors as:

“…**a significantly higher eGFR (by 9.87 ml.min/1.73 m^2^**)”

A correction has been made to the section [Section 7, page 7, paragraph 3]:

“**a significantly higher eGFR (by 9.87 ml/min/1.73 m^2^)”**

The original version of this article has been updated.

The text was incorrectly written as:

“…**17%–20% lower odds of** dementia of **mild cognitive impairment**, …”

A correction has been made to the section [Section 8, page 8, paragraph 2]:

“**17%–20% lower odds of** dementia or **mild cognitive impairment**, …”

The original version of this article has been updated.

The heading was incorrectly written as:

“**9 Vitamin K and insulin sensitivity and diabetes”**

A correction has been made to the section [Section 9, page 8, Heading]:

“**9 Vitamin K, insulin sensitivity, and diabetes”**

The original version of this article has been updated.

The text was incorrectly written as: “… **but the difference and the mechanism involved are not clearly understood.”**

A correction has been made to the section [Section 13, page 13, paragraph 1]:

“**… but the differences and mechanisms involved are not clearly understood**.”

The original version of this article has been updated.

**Citation correction**

Iwamoto et al. (118) was removed from the article. The citation has now been removed from [table 1, row 7] and section [Section 12, page 11, paragraph 1] and should read:

“Several randomized controlled trials (RCTs) demonstrated positive effects of vitamin K2 supplementation, particularly MK-4 and MK-7, on bone mineral density (116) and arterial elasticity (53).

The original version of this article has been updated.

